# Small intestine melanosis caused by clofazimine: A case report

**DOI:** 10.1097/MD.0000000000050332

**Published:** 2026-08-21

**Authors:** Wei-qiang Wang, Peng Li, Guang-ming Tang, Zhao Li, Zhong-min Chen

**Affiliations:** a Department of Gastroenterology, Central Hospital Affiliated to Chongqing University of Technology, Chongqing, China.

**Keywords:** clofazimine, melanosis, small intestine

## Abstract

**Rationale:**

Melanosis coli is a rare condition characterized by abnormal pigmentation of the gastrointestinal mucosa, most commonly involving the colon. Involvement of the small intestine is exceedingly rare, with only a few cases documented in the literature. Here, we present a case of small-intestinal melanosis and explore its potential pathogenesis and clinical significance.

**Patient concerns:**

A 40-year-old male presented with a 6-month history of abdominal pain and altered bowel habits. He had been taking clofazimine (100 mg/d) for pulmonary tuberculosis for 7 months. Notably, his symptoms and skin hyperpigmentation began 1 month after initiating clofazimine therapy. Total enteroscopy revealed diffuse gray pigmentation of the duodenal, jejunal, and ileal mucosa. Histological analysis showed lipofuscin-like granules resembling those observed in colonic melanosis. Methylene blue staining demonstrated uniform uptake, with brownish granules at the villous tips, producing a coral-like appearance. Histopathological examination confirmed the presence of chronic inflammation with mucosal pigment deposition.

**Diagnoses:**

Small-intestinal melanosis secondary to clofazimine therapy.

**Interventions:**

Clofazimine was discontinued, and no further treatment was administered.

**Outcomes:**

Three months after stopping clofazimine, the patient’s symptoms resolved, skin pigmentation faded, and follow-up enteroscopy showed normal mucosal color throughout the small intestine.

**Lessons:**

Intestinal melanosis is a rare disease that, in this case, may be related to long-term clofazimine use. Clinicians should consider this diagnosis in patients with unexplained intestinal pigmentation, particularly in those taking clofazimine. Further research is needed to elucidate its natural history and underlying mechanisms.

## 1. Introduction

Melanosis is a benign disease characterized by the accumulation of lipofuscin-like pigments within macrophages of the gastrointestinal mucosa. Melanosis coli is well documented and is primarily associated with the use of anthraquinone laxatives.^[1,2]^ However, intestinal melanosis remains extremely rare, with only a few reported cases confined to the terminal ileum^[3]^ or duodenum.^[4]^ Importantly, our case presented with a unique clinical presentation: colonoscopy revealed diffuse melanosis throughout the small intestine with complete sparing of the colon, a finding not previously documented in the literature.

The patient developed abdominal pain, altered bowel habits, and skin hyperpigmentation after taking clofazimine for 6 months, suggesting a possible causal relationship between this drug and the observed intestinal melanosis. Clofazimine is a special type of antibiotic used to treat leprosy and multidrug-resistant tuberculosis and is known to cause skin hyperpigmentation.^[5,6]^ Only sporadic case reports have been published regarding its association with duodenal or diffuse intestinal melanosis.^[7,8]^ This case highlights the endoscopic and histopathological features of clofazimine-induced intestinal melanosis and emphasizes the need for clinicians to be more aware of the potential risk factors in patients receiving long-term clofazimine therapy.

## 2. Case report

### 2.1. Chief complaints

A 40-year-old male presented with a 6-month history of abdominal pain accompanied by altered bowel habits.

### 2.2. History of the present illness

Six months earlier, he began experiencing intermittent, dull, moderate-intensity pain localized to the epigastric and periumbilical regions, predominantly occurring at night and frequently disrupting sleep. The pain was accompanied by increased stool frequency (2–4 times per day) and loose yellow stools and was consistently relieved by defecation.

### 2.3. History of past illness

The patient had a history of pulmonary tuberculosis and had been receiving clofazimine soft capsules (Shanxi Liye Pharmaceutical Co., Ltd., China; National Drug Approval Number H32021093; 100 mg/d) for 7 months. His gastrointestinal symptoms and skin pigmentation began approximately 1 month after initiating clofazimine therapy.

### 2.4. Personal and family history

The patient denied any family history of malignant tumors.

### 2.5. Physical examination

Vital signs: temperature: 36.8 °C; pulse: 76 beats per minute; respiratory rate: 18 per minute; blood pressure: 114/73 mm Hg. General examination: Generalized cutaneous hyperpigmentation was observed, ranging from yellow to brown, without evidence of jaundice or petechiae. Cardiopulmonary examination: Breath sounds were clear bilaterally with no rales. Heart sounds were normal, with a regular rhythm and no audible murmurs.

### 2.6. Abdominal examination

The abdomen was soft and non-distended with normal bowel sounds. Mild tenderness was observed in the epigastric and periumbilical regions. No hepatosplenomegaly or palpable masses were detected.

### 2.7. Laboratory examinations

The patient exhibited an elevated white blood cell count (11.59 × 10^9^/L) and mildly elevated blood glucose (6.21 mmol/L). Levels of tumor markers, including carcinoembryonic antigen, carbohydrate antigen-125, carbohydrate antigen-199, carbohydrate antigen-724, and alpha-fetoprotein, were within normal limits. Hepatitis B serology, amylase, lipase, D-dimer, coagulation profile, troponin, cardiac enzymes, electrolytes, and liver and renal function test results were all unremarkable. Stool analysis revealed no white or red blood cells, and the fecal occult blood test results were negative.

### 2.8. Imaging examinations

**Figure 1.**
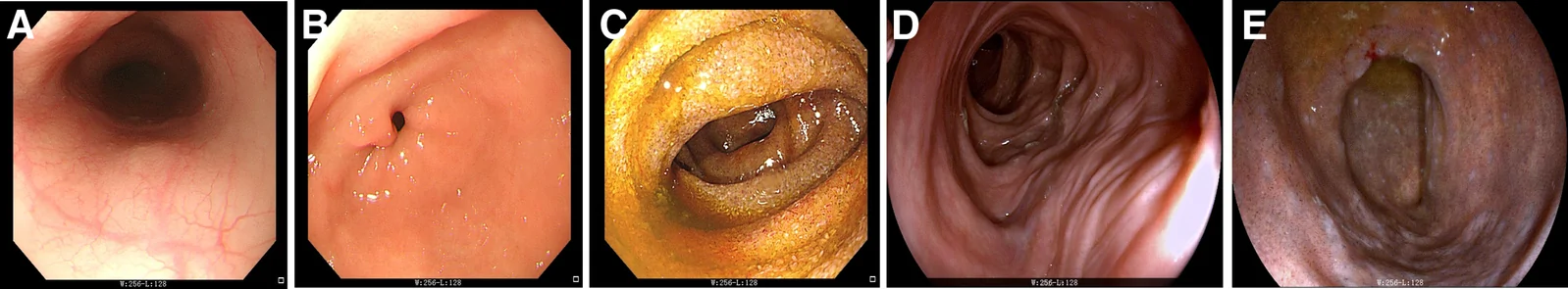
Upper endoscopy and colonoscopy. (A) Pink esophageal mucosa with clear vascular texture, no abnormalities; (B) orange-red gastric mucosa, smooth surface, no abnormalities; (C) gray duodenal mucosa with pigmentation; (D) pink mucosa in the colon and rectum, smooth surface, no abnormalities; (E) grayish-brown mucosa with pigmentation observed in the terminal ileum.

Upper endoscopy revealed normal findings in the esophagus and stomach. The esophageal mucosa appeared pink with a clear vascular pattern and no abnormalities (Fig. 1A). The gastric mucosa was orange-red with a smooth surface and no lesions (Fig. 1B). In contrast, the duodenal mucosa appeared gray and pigmented (Fig. 1C).

Colonoscopy revealed normal pink mucosa in the colon and rectum, with a smooth surface and no abnormalities (Fig. 1D). However, a grayish-brown pigmentation was observed in the terminal ileum (Fig. 1E).

### 2.9. Total enteroscopy

**Figure 2.**
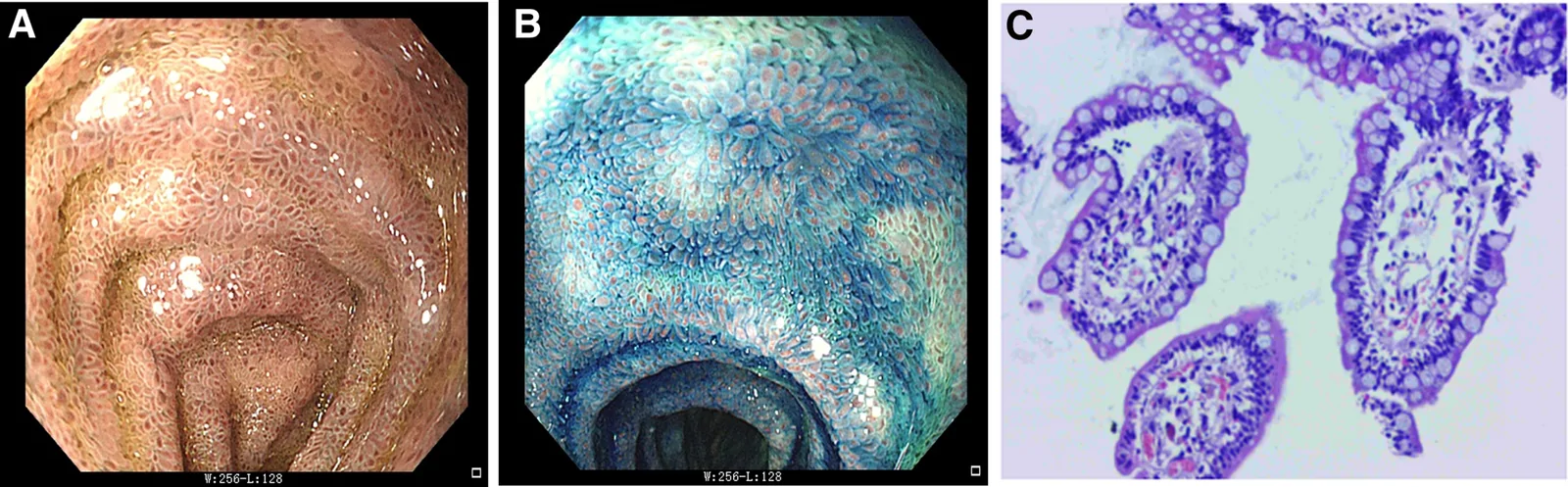
Total enteroscopy. (A) Diffuse gray pigmentation in the mucosa of the jejunum showing lipofuscin-like granules; (B) following methylene blue staining, the small intestinal mucosa was uniformly colored, with brownish granules visible at the villous tips, resembling coral; (C) histopathology check showed chronic inflammatory changes in the small intestinal mucosa with pigment deposition.

Diffuse gray pigmentation was observed throughout the mucosa of the duodenum, jejunum, and ileum. The mucosal surface was otherwise smooth, without evidence of erosion or ulceration, and histologically resembled melanosis coli (Fig. 2A). After methylene blue staining, the mucosa exhibited uniform coloration, with brownish granules concentrated at the villous tips, creating a coral-like appearance (Fig. 2B).

### 2.10. Histopathology

Microscopic examination of the small intestinal mucosa revealed chronic inflammatory changes, accompanied by pigment deposition within the lamina propria (Fig. 2C).

### 2.11. Diagnosis and management

A diagnosis of small-intestinal melanosis secondary to clofazimine therapy was established. Clofazimine was discontinued, and no further treatment was administered.

### 2.12. Outcome and follow-up

**Figure 3.**
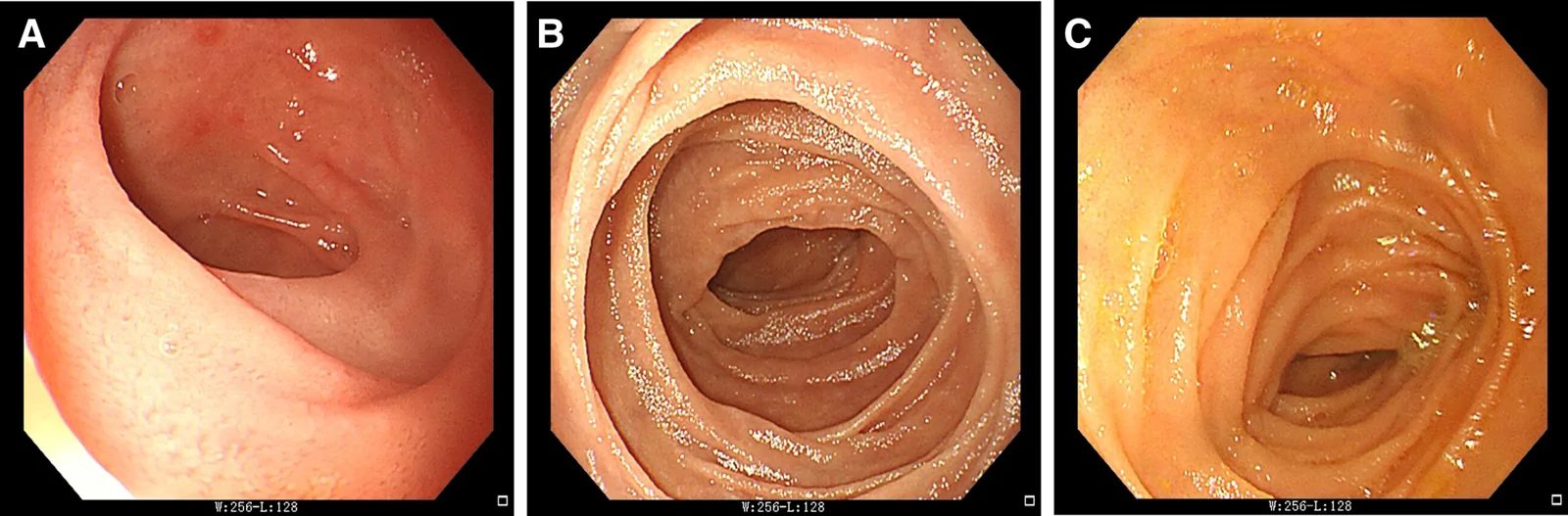
Repeat total enteroscopy showed complete resolution of the mucosal pigmentation at 3-month follow-up. (A) Duodenum; (B) jejunum; (C) ileum.

At the 3-month follow-up, the patient’s abdominal pain had resolved, bowel habits had normalized, and skin hyperpigmentation was gradually fading. Repeat total enteroscopy revealed that the mucosal coloration of the duodenum (Fig. 3A), jejunum (Fig. 3B), and ileum (Fig. 3C) had returned to a normal pink appearance, with no residual pigmentation observed.

All the above materials and enteroscopy findings were approved by the patient (informed consent obtained) and were published with the approval of the Ethics Committee of the Central Hospital Affiliated to Chongqing University of Technology.

## 3. Discussion

Small-intestinal melanosis is an exceedingly rare condition, with most reported cases linked to long-term clofazimine use.^[7]^ The clinical presentation of our patient, including chronic abdominal pain, altered bowel habits, and cutaneous hyperpigmentation, is consistent with previously documented cases.^[7]^ Notably, symptoms developed within 1 month of initiating clofazimine therapy, suggesting a potential dose- and duration-dependent relationship. Unlike melanosis coli, which predominantly affects the colon, melanosis involving the small intestine may lead to more significant motility disturbances due to the region’s critical absorptive and secretory functions.

Our case demonstrates the classic endoscopic features of small-intestinal melanosis: diffuse grayish-brown mucosal discoloration involving the duodenum, jejunum, and ileum; lipofuscin-like granular deposits; and a coral-like appearance following methylene blue staining (Fig. 2). Interestingly, no pigmentation was observed in the gastric or colonic mucosa. The reason for this selective involvement of the small intestine remains unclear.

The most likely cause of the patient’s small-intestinal melanosis was chronic clofazimine administration. This conclusion is supported by several observations: the patient had a prolonged history of clofazimine use; typical cutaneous pigmentation and gastrointestinal symptoms (abdominal pain and altered stool consistency) developed shortly after drug initiation; there was no history of other medications known to cause intestinal melanosis, such as anthraquinone-containing laxatives; and both symptoms and endoscopic findings resolved completely following the discontinuation of clofazimine.

The precise mechanism by which clofazimine induces small-intestinal melanosis remains unclear. Several potential explanations have been proposed. First, clofazimine’s lipophilicity and absorption profile may contribute to its selective involvement. Being highly lipophilic, clofazimine primarily binds to fat following oral administration. Because the small intestine is the primary site of fat absorption, unlike the stomach and colon, this may account for its selective involvement. Second, deposition of colored particles may contribute to pigmentation. A clofazimine suspension, a brownish-red to reddish-brown oily liquid, contains pigmented particles that may be phagocytosed by macrophages and subsequently deposited in the small intestinal mucosa, leading to pigmentation. Further studies are needed to determine whether clofazimine exerts direct structural or functional effects on the small-intestinal mucosal cells.

Melanosis coli has been associated with an increased risk of colorectal polyps and cancer,^[9,10]^ but it remains unclear whether small-intestinal melanosis carries a similar risk. In this case, the small intestinal mucosa returned to normal 3 months after discontinuation of clofazimine (Fig. 3), and no polyps or neoplasms were observed. These findings suggest that this reversible, drug-induced form of melanosis may not confer an increased risk of malignancy. However, larger long-term follow-up studies are needed to confirm this observation.

## 4. Conclusion

This report describes the clinical, endoscopic, and histopathological features of a rare case of diffuse small-intestinal melanosis associated with clofazimine use.

It underscores the importance of considering this diagnosis in patients receiving clofazimine who present with gastrointestinal symptoms and unexplained small-bowel pigmentation. These findings contribute to a broader understanding of the potential adverse effects of clofazimine beyond cutaneous pigmentation. Notably, in this case, the condition appeared to be fully reversible following drug discontinuation.

## Author contributions

**Data curation:** Wei-qiang Wang, Peng Li, Zhao Li.

**Funding acquisition:** Wei-qiang Wang.

**Investigation:** Zhao Li, Guang-ming Tang.

**Methodology:** Zhao Li.

**Project administration:** Wei-qiang Wang, Zhao Li.

**Resources:** Zhao Li.

**Supervision:** Zhong-min Chen.

**Writing – original draft:** Wei-qiang Wang, Guang-ming Tang.

**Writing – review & editing:** Wei-qiang Wang, Zhong-min Chen.

## References

[1] Chiba T Wang T Kikuchi S. Colonoscopic resolution of melanosis coli after cessation of senna laxative use. Int Med Case Rep J. 2024;17:783–7. 39282237 10.2147/IMCRJ.S475869 PMC11402352

[2] Katsumata R Manabe N Monobe Y. Development, disappearance, and clinical course of melanosis coli: sex differences in the progression of severity. Acta Med Okayama. 2023;77:57–64. 36849146 10.18926/AMO/64362

[3] Batistatou A Panelos J Agnantis NJ. Melanosis intestini: case report. Diagn Pathol. 2006;1:3. 16759412 10.1186/1746-1596-1-3 PMC1459274

[4] Rana NK Minhas U Mahl T. A rare case of duodenal melanosis: case report. Cureus. 2020;12:e10475. 33083177 10.7759/cureus.10475 PMC7567319

[5] Karnan A Ledwani A. Clofazimine-induced skin pigmentation. Pan Afr Med J. 2024;47:86. 38737223 10.11604/pamj.2024.47.86.42513 PMC11087286

[6] Ito M Watanabe F Furuuchi K. Quantifying the reversibility of clofazimine-induced pigmentation in a patient with Mycobacterium abscessus pulmonary disease. Intern Med. 2025;64:1884–7. 39566985 10.2169/internalmedicine.4498-24 PMC12241785

[7] Zhang H Peng Q Zhao X He Y Liu J. Clofazimine enteropathy: a case of pigmentation of the whole small intestine caused by clofazimine. Endoscopy. 2024;56:E578–9. 38959978 10.1055/a-2344-8244 PMC11221920

[8] Bhangale NP Abraham P Joshi A Desai D. Clofazimine-related duodenal pigmentation. Indian J Gastroenterol. 2021;40:347–8. 33417175 10.1007/s12664-020-01128-6

[9] Wang Y Li L Niu X Gao F Chai N Linghu E. Melanosis coli: a contrast effect or an oncogenic effect? A large-scale retrospective cohort study. Int J Colorectal Dis. 2023;38:63. 36884096 10.1007/s00384-023-04357-1

[10] Katsumata R Manabe N Monobe Y. Severe grade of melanosis coli is associated with a higher detection rate of colorectal adenoma. J Clin Biochem Nutr. 2022;71:165–71. 36213792 10.3164/jcbn.22-19 PMC9519422

